# Mass Spectrometry-Based Untargeted Approaches to Reveal Diagnostic Signatures of Male Infertility in Seminal Plasma: A New Laboratory Perspective for the Clinical Management of Infertility?

**DOI:** 10.3390/ijms24054429

**Published:** 2023-02-23

**Authors:** Mariaimmacolata Preianò, Serena Correnti, Tahreem Arshad Butt, Giuseppe Viglietto, Rocco Savino, Rosa Terracciano

**Affiliations:** 1 Department of Health Sciences, Magna Græcia University, 88100 Catanzaro, Italy; 2 Department of Experimental and Clinical Medicine, Magna Græcia University, 88100 Catanzaro, Italy; 3 Department of Medical and Surgical Sciences, Magna Græcia University, 88100 Catanzaro, Italy

**Keywords:** mass spectrometry, proteome, seminal plasma, male infertility, seminal fluid, biomarker, laboratory medicine

## Abstract

Male infertility has been recognized as a global health problem. Semen analysis, although considered the golden standard, may not provide a confident male infertility diagnosis alone. Hence, there is the urgent request for an innovative and reliable platform to detect biomarkers of infertility. The rapid expansion of mass spectrometry (MS) technology in the field of the ‘omics’ disciplines, has incredibly proved the great potential of MS-based diagnostic tests to revolutionize the future of pathology, microbiology and laboratory medicine. Despite the increasing success in the microbiology area, MS-biomarkers of male infertility currently remain a proteomic challenge. In order to address this issue, this review encompasses proteomics investigations by untargeted approaches with a special focus on experimental designs and strategies (bottom-up and top-down) for seminal fluid proteome profiling. The studies reported here witness the efforts of the scientific community to address these investigations aimed at the discovery of MS-biomarkers of male infertility. Proteomics untargeted approaches, depending on the study design, might provide a great plethora of biomarkers not only for a male infertility diagnosis, but also to address a new MS-biomarkers classification of infertility subtypes. From the early detection to the evaluation of infertility grade, new MS-derived biomarkers might also predict long-term outcomes and clinical management of infertility.

## 1. Introduction

Infertility is a global health issue defined by the inability to conceive after 12 months or more of regular unprotected sexual intercourse [1]. It affects approximately 15% of reproductive-aged couples worldwide and a contributing male factor may be found in about half of the cases, either alone or in combination with female causes [1,2,3].

To date, semen analysis is the cornerstone for the routine evaluation of male fertility with WHO guidelines providing the basis for the standardization of laboratory procedures and reference values worldwide [4,5].

However, although providing valuable information, semen analysis alone is not sufficient to accurately assess male fertility potential or to distinguish fertile subjects from infertile ones [3,6,7]. Moreover, standard seminal analysis fails to properly identify etiological factors and to unravel the molecular and pathophysiological basis of male reproduction diseases. Male fertility disorders require a more in-depth analysis, especially in the case of unexplained male infertility, a condition with unknown etiology which affects roughly 30% of men with normal semen parameters [3,6,8,9,10]. Other advanced sperm function tests such as sperm DNA damage and oxidative stress assays were recently introduced in routine clinical evaluations to predict reproductive outcomes in a more accurate way [4]. However, these additional screening tools still fail to explain the underlying mechanisms at a subcellular level that are associated with the different infertility phenotypes [11,12].

Hence, in the era of precision medicine, ‘omics’ technologies are in constant development, exploring for new, reliable and disease-specific biomarkers, with the aim of improving the diagnosis and prognosis of male fertility disorders and to select the best fitting therapeutic actions.

In this scenario, proteomics has emerged as an important tool for providing insights into the underlying molecular processes associated with male infertility. Proteomics technologies could overcome gaps in information from standard semen analysis and other sperm quality tests with limited diagnostic value [13,14].

The progress in research technology and techniques in the field of proteomics led to the development of innovative and reliable platforms for the non-invasive biomarker-based male infertility diagnosis. The improved resolution, sensibility and accuracy of the mass spectrometry (MS) platforms allowed the high-throughput characterization of proteins associated with male fertility disorders [15,16].

In recent years, due to the presence of tissue-specific molecular mediators, an increased amount of MS-based proteomics investigations was carried out on semen and seminal plasma (SP) to better identify disease-specific biomarkers of male infertility [14,17,18,19,20,21].

In particular, the characterization of proteome profile of SP by MS-based approaches, has revealed its key role in reproductive processes and its potential as a screening, diagnostic and prognostic instrument in male fertility assessment [22,23,24,25,26,27,28,29,30,31,32,33,34,35,36,37]. In fact, SP that for a long time was considered only a passive medium for spermatozoa transport and protection is highly enriched in proteins, RNAs, lipids and other metabolites. All these molecules exert important effects over sperm function and male fertility, capturing growing interest for their potential as clinical samples for non-invasive diagnostics [14,17,38].

The huge amount of MS-based proteomic data subjected to bioinformatic analysis has provided extensive examination about distribution, molecular and functional analysis for the SP candidate biomarkers [13,39].

This review provides an overview of the main MS-based untargeted approaches for comparative proteomics between fertile and infertile men categorized according to their quantitative and/or qualitative alteration of seminal parameters. In particular, key MS techniques and strategies adopted (bottom-up or top-down) to analyze the SP proteome with a rapid overview on the major proteomics findings and candidate biomarkers for male infertility diseases are highlighted. Challenges and limitations of these approaches, which could accelerate the development of new MS laboratory diagnostics applications in the area of clinical management of male infertility, will be discussed.

## 2. Semen and Seminal Plasma

### 2.1. Human Semen and Its Composition

Semen is a complex body fluid, which contains a heterogeneous mixture of components produced by different sex accessory glands, including the seminal vesicles, the prostate gland and the bulbourethral glands [40].

Semen is released during ejaculation and is composed of two major fractions: the cellular and the fluid fractions. The cellular fraction is composed of spermatozoa, which are produced in the testes and stored in epididymides, while the fluid fraction contains different liquid secretions, which contribute to generate the SP. Spermatozoa accounts only for approximately the 5% of the whole semen volume, while SP represents the remaining 95% [17].

Immediately after ejaculation, human semen appears as a thick fluid, with a gelatinous structure, referred to as the coagulum, which traps and immobilize the spermatozoa. This coagulum is typically liquefied within 15–30 min, mainly by the activity of the prostate-specific antigen (PSA or KLK3), a chymotrypsin-like serine protease, which hydrolyzes highly abundant proteins forming the coagulum (Semenogelins I and II, fibronectin). These proteolytic cleavages allow the liquefaction of the seminal clot and the increase in spermatozoa motility, which are able to reach the female reproductive tract [41,42]. Other enzymes which participate in the semen liquefaction include KLK2, KLK5 and KLK14, which have been reported to hydrolyze fibronectin and Semenogelins in ex vivo and in vitro studies, and also KLK6, KLK7 and KLK13, which showed catalytic capacity toward fibronectin [43].

### 2.2. Features of Human Seminal Plasma

The difference between semen and SP lies in the fact that SP is only the fluid portion of the entire ejaculate, while semen comprises both the spermatozoa and SP.

**Figure 1:**
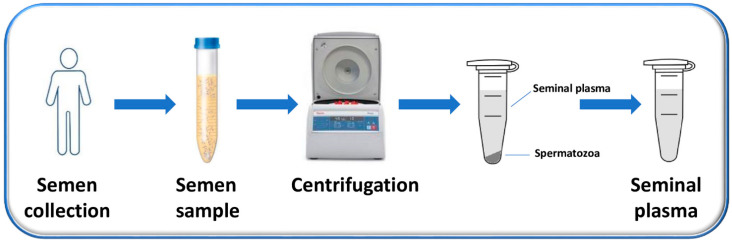
Flow chart of the process used to obtain SP. Semen is collected from men by masturbation into sterile containers after (3–5) days of sexual abstinence and is allowed to liquefy. After liquefaction, semen samples are centrifuged to separate the supernatant (representing the SP) from the pellet (composed mainly by the sperm cells).

SP is the supernatant, easily obtained after the centrifugation of the liquefied semen and the removal of sperm cells and cell debris, which constitute the pellet (Figure 1) [44].

It has a very heterogeneous and complex molecular composition, including lipids, glycans, inorganic ions, metabolites, cell free DNA, RNA, microRNAs, peptides, proteins and oligosaccharides. It contains secretions derived from multiple glands of the reproductive tract, among which seminal vesicles and prostate are the main contributors in terms of volume (~65% and ~25%, respectively). In particular, secretions from seminal vesicles are highly enriched by cytokines, prostaglandins and fructose, sources of energy for spermatozoa [45], while prostate glands secrete a fluid constituted by proteolytic enzymes, citrate, lipids, calcium, magnesium and zinc [46,47]. These secretions also include basic polyamines, which warrant an alkaline environment to the semen, contributing to the survival of sperm cells in the acidic milieu of the vagina.

A minor contribution of the seminal fluid volume is represented by the testis and epididymis (~10%), and finally, by bulbourethral (Cowper) and periurethral (Littre’s) glands (~1%). Their secretions also act as lubricants of the semen, allowing a more efficient sperm transfer [48].

SP also contains a large number of extracellular vesicles (EVs) which have heterogeneous dimensions, origin, and “molecular cargo” and are a rich source of proteins [49]. They are especially secreted by the epididymis (epididymosomes) and the prostate (prostasomes) and are implicated in promoting spermatozoa motility, immunomodulation, antibacterial activity and antioxidant protection [50]. Several studies investigated the proteome of the EVs, collecting them by semen centrifugation, the removal of spermatozoa and different ultracentrifugation steps of the supernatant. This allows for exclusively analyzing the proteome associated with these extracellular vesicles [51,52,53,54,55].

Most of the studies on human SP have been focused on the whole SP [25,26,27,28,29,30,31,32,33,34,35,36,37], whose analysis can be affected by the presence of the EVs.

Bianchi et al. performed the first functional proteomic study on the vesicle-free (vf) soluble fraction of human SP in normozoospermic healthy donors by combining two-dimensional gel electrophoresis (2-DE), matrix-assisted laser desorption/ionization-time-of-flight (MALDI-TOF) MS and bioinformatic tools for pathway analyses [22]. Proteomic data demonstrated that vf-SP includes a very limited set of highly abundant unique proteins subjected to massive co- and/or post-translational modifications. Functional analysis showed that these proteins were mainly involved in catalytic activity, immune response, apoptotic process, seminal clot regulation, and central nervous system development and morpho-functional maintenance. These results demonstrated that vf-SP represents a catalytic milieu of proteases and their inhibitors which play a key role in semen coagulation and liquefaction. Data analysis also revealed that some vf-SP proteins are probably involved in the modulation of structural and functional properties of spermatozoa during sperm migration in the male and female genital tracts [22].

### 2.3. Importance and Advantages of SP as Biomarker Source of Male Reproductive System Disorders

SP contains numerous proteins, with a concentration that ranges between 35 and 55 mg/mL, making it an accessible source for proteins identification [17,23,44]. Proteomic and functional studies have highlighted the physiological roles of SP proteins. They mediate important functions not only on the sperm activity including metabolism, maturation, motility and capacitation, but also on semen coagulation, liquefaction and fertilization. They are also involved in delivering and providing nutrition to spermatozoa during their travel throughout the male and female reproductive tracts, in the increase of the immune response, interaction with the zona pellucida and modulation of the acrosome reaction [14,56]. All these functions are crucial for natural reproductive success.

Because of its functional characteristics, SP reflects the local pathophysiology of the male reproductive system, thus representing an optimal and promising resource for the discovery of biomarkers of male infertility and other related disorders [14,38]. In fact, being located close in proximity to the male reproductive tract, it is highly enriched of specific proteins and peptides, that have a better predictive value as biomarkers for the study of these pathologies than other markers located in serum or urine, where they are much more diluted and less expressed. Therefore, it is easier to identify and quantify them in seminal plasma by analytical techniques [44].

For these reasons, in recent years, SP has gradually captured interest for its promising potential as a clinical sample for non-invasive diagnostics. In fact, as previously outlined in Figure 1, SP is obtained by a non-invasive and safe procedure, based on the simple centrifugation of the semen, collected by masturbation into sterile containers after (3–5) days of sexual abstinence.

The discovery of infertility biomarkers in human SP and their use in the clinical setting, might offer a powerful and reliable approach for overcoming and replacing currently invasive surgical techniques and blood or urine-based tests, which have limitations, including a lack of specificity and prognostic significance [44]. SP biomarkers for male reproductive system disorders could also outperform traditional semen analysis and sperm functional tests, which have a poor diagnostic performance and cannot precisely and accurately predict the fertility status of a man alone [6,38].

### 2.4. Assessment of Proteolytic Activity in SP by MS

The human semen liquefaction process is controlled by different factors, including proteases and protease inhibitors, which represent a high percentage of all the proteins identified in human SP [14]. The high number of these components highlights the importance of this system in this body fluid.

It is well known that human semen liquefaction is a proteolytic process, occurring after ejaculation and mainly requiring the enzymatic activity of the PSA, also known as kallikrein-related peptidase 3 (KLK3), which changes semen from a gel-like coagulum to a more fluid consistency. This process is fundamental for the spermatozoa to gain their motility and reach the fertilization site in fallopian tubes [43]. There are also other members of the KLKs family which participate in the process, including KLKs 2, 3, 4, 5, 8, 11, 12, and 14 that are secreted by the prostate and act in a protease cascade.

Semen liquefaction is also controlled by endogenous inhibitors such as Zn^2+^, secreted in the prostatic fluid, that maintain KLK3 in an inactive form. After ejaculation, prostatic fluids are combined with seminal vesicle fluids, containing semenogelins, which sequester Zn^2+^, thus activating KLK3. This performs site-specific cleavages of semenogelins into low molecular weight peptides, determining the dissolution of the coagulum [43].

Residual proteolytic activity if still present in SP might represent one of the major issues in the context of proteome analysis.

Robert and colleagues [57] reported the addition of a protease inhibitor cocktail (PIC) containing 4-(2-aminoethyl) benzenesulfonyl fluoride hydrochloride (AEBSF), a serine protease inhibitor, that blocked PSA activity, in human SP. They demonstrated that, when PSA was treated with the protease inhibitor, the degradation and the hydrolysis of semenogelins were prevented, supporting the notion that PSA is the main semenogelins processing enzyme in the early stage of semen liquefaction.

In a very recent study, Correnti et al. [36] investigated the stability of human SP samples, by assessing the variation of the total peak numbers between samples with and without PIC at several time points by MALDI-TOF MS analysis. More precisely, PIC was added to samples after semen liquefaction at 0 and after 60, 90, 120 and 150 min at room temperature and after 1 and 120 days of storage at −80 °C. The samples were then analyzed by MALDI-TOF MS. They observed no statistically significant variation in peaks number neither up to 2.5 h at room temperature nor up to 120 days of storage at −80 °C, and also, no statistically significant difference in peaks number was observed between samples with and without the use of PIC. These data demonstrated that SP samples are stable for at least 2.5 h at room temperature and for at least 4 months when stored at −80 °C and that no residual proteolytic activity is still present in SP after the liquefaction of coagulum [36].

## 3. MS Untargeted Approaches for Differential Proteomics Analysis between Fertile and Infertile Subjects

**Figure 2:**
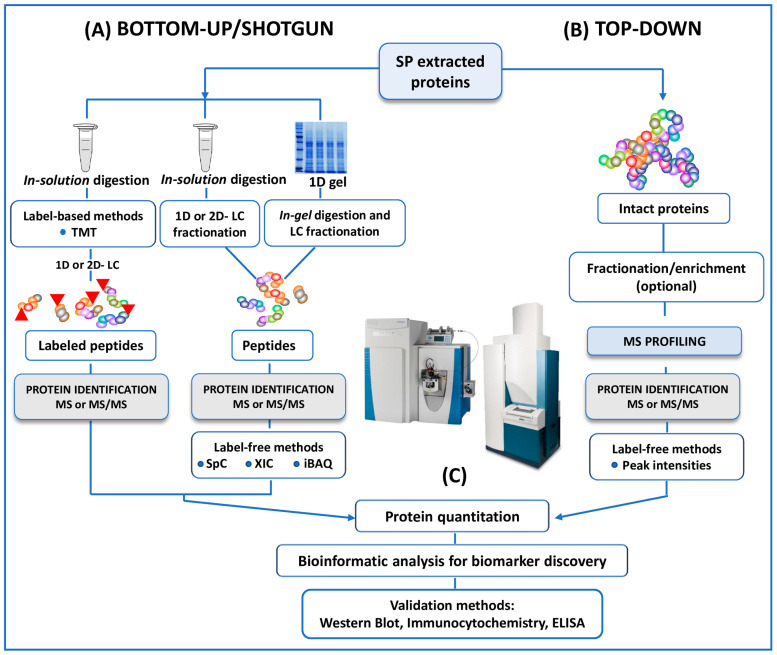
A schematic outline of all the protocols here reviewed in both bottom-up and top-down untargeted MS approaches for the proteomic analysis of human SP. (**A**) In conventional bottom-up (or shotgun) strategy, proteins extracted from SP are reduced, alkylated and digested in solution. The resulting proteolytic fragments are fractionated by 1D- or 2D- LC before MS analysis. In a one more classical approach, included in the category of the bottom-up strategies, SP proteins are separated by 1D (here reviewed) or 2D SDS-PAGE then the bands (from 1D) or the spots (from 2D) of interest are enzymatically cleaved by the mean of “*in gel* digestion” protocols. Protein quantitation, when requested, could be performed by labeling strategies (iTRAQ or other TMT strategies). In this case, proteolytic peptides are labeled with isobaric mass tags and pooled together prior to HPLC separation. Multiplexed quantitation of changes in SP protein expression is measured by MS and MS/MS experiments. MS-quantitation in several studies is assessed alternatively by label-free methods (SpC, XIC or iBAQ). (**B**) In the top-down investigations here reviewed no digestion experiments are performed. The mixture of intact proteins from SP is fractionated, or enriched and/ or fractionated before MS or MS/MS analysis. The quantification is performed by label-free methods in the studies. (**C**) Identified (and or quantified) proteins are analyzed by bioinformatics tools for the extrapolation of differentially expressed proteins as candidate biomarkers of male infertility. Putative biomarkers from MS analysis are then validated using different approaches, including Western Blot, immunocytochemistry, ELISA, Selected reaction monitoring/Multiple Reaction Monitoring.

MS has emerged as a key platform for proteomic analyses of human SP, with two main approaches referred to as ‘bottom-up’ (also known as shotgun) and ‘top-down’ proteomics (Figure 2). Most of the studies here reviewed used a bottom-up approach to study the differential expression of SP proteins between fertile and infertile men [25,26,27,28,29,30,31,32,33,34,35], while top-down approach is reported in only two investigations [36,37].

Briefly, in the bottom-up studies, whole proteins extracted are digested into peptides using trypsin (in-solution digestion). Proteolytic cleavage products are then fractionated by mono-dimensional (reverse phase) or bi-dimensional (strong-cation-exchange or strong-anionic-exchange followed by reverse phase) liquid chromatography (LC), and then fractions of peptides are analyzed by MS or MS/MS (Figure 2) [25,26,27,28,30,31,32,33,34,35]. In a more classical approach, extracted proteins are separated by 1D-SDS-PAGE. The bands (1D) of interest are then proteolytically digested (in-gel digestion) and analyzed by LC coupled to MS/MS (LC-MS/MS) (Figure 2) [29].

Unlike the bottom-up strategy, top-down studies [36,37] enable the analysis of intact proteins and peptides naturally occurring in the proteomes, avoiding any enzymatic digestion and therefore sample alteration (Figure 2). MALDI- and SELDI-TOF MS, HPLC-MS and MS/MS analysis of intact proteomes or sub-proteomes can be included in this category [36,58]. The investigation of intact proteins (rather than enzymatically digested peptides) by MS allows for the better characterization of the protein state. In particular, the use of top-down approaches enables the detection of the biologically active forms of the proteins, the location and identity of post-translational modifications (PTMs). Substantially, the top-down strategy provides a deeper understanding of specific protein isoforms (proteoforms), which are not easily detected by standard protein profiling techniques (Figure 2) [58].

### 3.1. SP Proteome Analysis by Bottom-Up Approach

The first large-scale proteomic analysis from a single sample of human SP was performed by Pilch and Mann by 1D-PAGE in combination with LTQ-FTICR MS in order to extensively characterize the SP proteome providing better insights into sperm functions [23]. The preliminary protocol adopted in this study before MS analysis was based on the use of 1D-PAGE without any further biochemical purification steps. This choice implied no loss of both hydrophobic and highly charged proteins before MS analysis, achieving significantly better protein coverage. In fact, 923 SP proteins were identified with high confidence by this approach. Protein expression data were further analyzed by the GoMiner program package to assign their cellular localization, molecular functions and biological processes [23]. This study revealed the presence of a large number of extracellular proteins and proteins specific of male accessory glands with a key role in spermatozoa survival. Interestingly, the highly confident proteomic data originated from this investigation, provided an inventory of SP proteins that has served as a reference for SP proteomics studies with a special focus on male infertility and fertilization, and testicular and prostatic cancers.

Milardi and colleagues implemented an extensive analysis of human SP in fertile subjects (*n* = 5) by the use of high-resolution LTQ-FT MS technology [59]. In this study, 1487 unique proteins per single sample were identified by performing the *in-solution* digestion of the individual samples followed by HPLC using a C18 column before LTQ-FT MS. Additionally, the authors also identified 83 common proteins in all fertile men (such as semenogelin I and II), obtaining the panel of proteins involved in reproduction regardless of interindividual variability.

Drabovich and colleagues performed a multi-step strategy to assess biomarker identification for the differential diagnosis of azoospermia [25]. In particular, SP samples from normal fertile men (*n* = 12), infertile men with proven non-obstructive azoospermia (*n* = 10) and previously fertile men who had undergone a vasectomy (*n* = 8) (which simulated obstructive azoospermia) were analyzed by using LC-ESI-triple-quadrupole and ion-trap/Orbitrap MS (Table 1). First, a multiplex label-free selected reaction monitoring (SRM) assay was performed to measure the relative abundance of 31 proteins in the unfractionated digest of both pooled samples (5 normal, 5 non-obstructive azoospermic and 5 post-vasectomy) and 30 individual SP samples. SRM is a quantitative targeted proteomic assay, which is most commonly performed on a triple-quadrupole and allows for the measurement of multiple proteins in a single assay, thanks to the multiplexing capabilities [60]. Of the 31 proteins measured, 18 showed a statistically significant difference in disease samples compared to normal samples. To validate these 18 candidate biomarkers, heavy isotope-labeled internal standards were synthesized to once again assess their concentrations in the same cohort of 30 individual samples. In conclusion, they proposed a panel of biomarkers able to differentiate between normal, non-obstructive azoospermia and post-vasectomy (which simulated obstructive azoospermia). Among these, the most promising candidates were testis expressed 101 (TEX101), lactate dehydrogenase C (LDHC), sperm associated antigen 11B (SPAG11B), prostaglandin D2 synthase (PTGDS), mucin 15 (MUC15), and protein FAM12B, which were down-regulated in non-obstructive azoospermic and post-vasectomy men (Table 2).

In 2013, the same group focused on the previous 18 biomarker candidates to select the minimal number of markers necessary for the differential diagnosis of azoospermia [26]. In total, 119 SP samples (42 men with normal spermatogenesis, 25 men with non-obstructive azoospermia, and 52 with obstructive azoospermia/postvasectomy men) were analyzed using the above-mentioned MS-based multiplex SRM assay (Table 1). Two proteins (epididymis-expressed ECM1 and testis-expressed TEX101) were identified for the differential diagnosis of azoospermia. In particular, extracellular matrix protein 1 (ECM1) was increased in fertile and non-obstructive azoospermic men and decreased in obstructive azoospermia, while TEX101 was increased in fertile men and decreased in non-obstructive azoospermic and obstructive azoospermic men. These data were validated by ELISA and immunohistochemistry (Table 2). In summary, this approach provided a two-biomarker decision tree for the non-invasive differential diagnosis of non-obstructive azoospermia and obstructive azoospermia and for the differentiation of non-obstructive azoospermia subtypes.

Del Giudice et al. used LC-ESI- quadrupole-Orbitrap MS in order to determine SP biomarkers of testicular function in adolescents with varicocele [27]. In particular, they compared the proteomic profiles among three main groups by a label-free shotgun approach. In the control group, 23 subjects were recruited without varicocoele and with normal semen analysis. The other two groups were formed by patients with varicocoele and normal semen analysis (37 subjects) and by patients with both varicocoele and altered semen analysis (17 subjects) (Table 1) [27]. For each group, the samples were pooled, trypsin digested and analyzed by LC-ESI-quadrupole-Orbitrap MS. A total of 541 proteins were identified and a label-free quantification approach was performed using intensity-based absolute quantification (iBAQ). Briefly, iBAQ provides protein quantification as a fraction between the sum of peak intensities of all peptides matching to a specific protein by the number of theoretically observable peptides [61]. The authors proposed calcium-binding protein (Cab45), left–right determination factor 1 (protein lefty-1), deoxyribonuclease-1 (DNase I) and lipid phosphate phosphohydrolase 1 (PAP2-alpha) as candidate biomarkers of spermatogenesis and homeostasis associated to the control group, while insulin-like growth factor-binding protein 7 (IBP-7), Ig gamma-3 chain C region (HDC) and cysteine rich secretory protein 3 (CRISP-3) as biomarkers associated to varicocoele groups (Table 2). Among the suggested biomarkers selected by a multivariate statistical analysis, confirmatory results were obtained for two proteins using western blot: Cab45, that was underexpressed in varicocoele groups and CRISP-3, that was overexpressed in adolescents with varicocoele and altered semen analysis (Table 2).

Sharma et al. used LTQ linear ion trap MS coupled to ESI ion source, in a label-free bottom-up approach, with the aim to compare the protein expression of SP samples derived from 26 normozoospermic men, 22 teratozoospermic, 6 oligozoospermic and 10 oligoteratozoospermic patients (Table 1) [28]. SP samples were pooled into four groups, according to subject category, and precipitated in cold acetone in order to improve the protein recovery in the sample. Then samples were subjected to in-solution digestion and to the LC-MS/MS system. The authors identified 35 proteins and performed a label-free quantification approach using spectral counting (SpC) (Table 1). In this approach, the protein quantification is determined by counting the total number of MS/MS spectra of all the peptides derived from the same protein [62]. Out of the identified proteins, 20 were differentially expressed among the four groups (Table 2). For example, the semenogelin I (SEMG I) isoform b preprotein was upregulated in the oligoteratozoospermic group; clusterin (CLU) isoform 1 was downregulated in the oligozoospermic group; prostatic acid phosphatase (PAP) was downregulated, and protein DJ-1 was overexpressed in the teratozoospermic and oligozoospermic groups (Table 2). Curiously, at the same time, DJ-1 (a protein involved in stress response) was found to be underexpressed in oligoteratozoospermic patients; this contrasting result requires further investigations. The functional bioinformatic analysis demonstrated that biological regulation is the mainly affected process and revealed that the identified proteins are mostly of extracellular origin (Table 2). However, it should be taken into account that the authors did not report any kind of normalization for protein concentration, and this could represent an important pre-analytical bias, potentially affecting the validity of the obtained data, especially in terms of differentially expressed proteins (Table 1).

The hybrid ion trap/Orbitrap mass spectrometer was used by Wang et al. to compare the SP proteome between 20 healthy control donors and 38 asthenozoospermic men [29]. SP proteins were first separated by 1D-SDS PAGE, gel bands were then digested, and peptides were analyzed by the LC-MS/MS (Table 1) [29]. In total, 741 proteins were identified and quantified using the SpC method; among these, 45 proteins were upregulated and 56 were downregulated in asthenozoospermic men compared to control subjects (Table 2). The majority of these proteins derive from the epididymis and prostate, suggesting that functional abnormalities of the epididymis and prostate can contribute to asthenozoospermia. The authors proposed intelectin-1 (ITLN1), alcohol dehydrogenase (ADH), delta-aminolevulinic acid dehydratase (ALAD) and DJ-1 as potential biomarkers for oxidative stress in asthenozoospermic patients; in particular they suggested that the downregulation of DJ-1, validated by western blot analysis, lead to oxidative stress, affecting the quality of semen in asthenozoospermia (Table 2). On the contrary, as mentioned previously, Sharma et al. [28] found an over-expression for DJ-1 in teratozoospermic and oligozoospermic patients, while a down-regulation in the case of oligoteratozoospermic ones was observed. These apparently contrasting findings require further investigations to better elucidate the role of this potential biomarker.

The same MS technology was used also by Batruch and colleagues to compare the SP proteome of fertile control men (*n* = 5), non-obstructive azoospermic patients (*n* = 5) and post-vasectomy/obstructive azoospermic patients (*n* = 5) (Table 1) [30]. SP samples were pooled, digested, fractionated by SCX chromatography followed by reversed-phase (RP) LC and analyzed by LTQ-Orbitrap MS. In SCX-RP LC, proteins are first separated on the basis of their charge (more specifically on the basis of the interaction between charged groups of the analyte and the negatively charged stationary phase) [63,64], and then on the basis of their hydrophobicity by using reverse phase columns. The most commonly used columns are the C18, silica-based columns derivatized with alkane chain containing 18 carbons to generate a hydrophobic surface [63].

A total of 2048 proteins were identified and quantified by Batruch et al., using two label-free quantitative approach (SpC and extracted ion chromatograms -XIC) [30]. The XIC methods enable to quantify proteins or peptides measuring the signal intensity, *m/z* values and the retention time of ions from chromatograms obtained in LC-MS measurements for specific peptides [65]. By this approach, the authors obtained candidate biomarkers useful to discriminate non-obstructive and obstructive azoospermia; they found 34 proteins up-regulated and 18 down-regulated in controls compared to non-obstructive azoospermic men, 59 up-regulated and 16 down-regulated in non-obstructive azoospermic men compared to post-vasectomy/obstructive azoospermic patients. Some of these proteins are shown in Table 2.

Herwig et al. performed a label-free bottom-up approach in order to compare the proteomic profile of SP from 11 pooled fertile and 11 pooled infertile men with oligoasthenoteratozoospermia, using a hybrid linear ion trap/quadrupole/Orbitrap mass spectrometer (Table 1) [31]. Pooled SP samples were trypsin digested and analyzed by the hybrid linear trap/quadrupole/Orbitrap mass spectrometer. Using SpC and Gene Ontology (GO) functional annotation, 46 proteins were identified, among which 24 proteins were found to be upregulated in the infertile compared to fertile men. These proteins are mainly involved in metabolism and inflammation, defense, and stress responses, suggesting their influence on infertility, particularly due to oxidative stress. In particular, α-1-antichymotrypsin (AACT) and aldose reductase (ALDR) were upregulated in oligoasthenoteratozoospermic patients (Table 2).

Wu et al. used a quantitative bottom-up proteomics approach to identify and quantify SP proteins in three normozoospermic and three asthenozoospermic men (Table 1) [32]. SP samples were treated with an acetone solution buffer over night for the precipitation and recovery of the proteins. Then, SP samples were digested, and the resulting peptides were labeled using the tandem mass tag (TMT) strategy. The peptide mixture was analyzed using a LTQ Orbitrap Velos mass spectrometer. TMTs are isobaric tags which label the peptides targeting the N-terminal position. These tags are fragmented by the MS/MS process and generate reporter ions, whose amount is directly proportional to the amount of the labeled peptides in the samples and is used to obtain quantitative information [66] (see also Figure 2). Thanks to this strategy, the authors were able to identify a total of 524 proteins. The biological functions and origins of these proteins were also determined by the integration of different proteomic datasets and bioinformatics databases. Finally, they found 29 differentially expressed proteins between the two analyzed groups. Bioinformatic analysis revealed that most of these proteins are mainly associated to sperm motility and male infertility, suggesting their potential role as biomarkers of asthenozoospermia. Validation analyses, using western blot, were performed for four proteins (KLK2, HSPA2, SORD, ANAX2), which confirmed the up-regulation of heat shock protein family A (Hsp70) member 2 (HSPA2) and the down-regulation of kallikrein related peptidase 2 (KLK2), sorbitol dehydrogenase (SORD), annexin A2 (ANAX2) in asthenozoospermic compared to normozoospermic (Table 2). However, an important limitation was the small pool of participants which compromised the significance of the study and the robustness of the identified biomarkers (Table 1).

In another study, Barrachina and colleagues used a bottom-up approach to analyze SP samples from four normozoospermic, four asthenozoospermic, four oligozoospermic and four azoospermic men by LC-ESI-ion trap/ORBITRAP MS (Table 1) [33]. In particular, SP samples were precipitated in cold acetone to improve protein recovery and then trypsin digested. Resulting peptides were labeled with TMT isobaric tags prior to LC-MS analysis. Proteomic data were analyzed by standard statistical analyses of relative protein quantification values (ANOVA and Pearson correlation test) which revealed a set of six differentially expressed proteins, correlated with sperm concentration. These proteins included cysteine rich secretory protein 1 (CRISP1), epididymal secretory protein E1 (NPC2), serine peptidase inhibitor, Kunitz type 3 (SPINT3), and ECM1 that were down-regulated in patients with low or an absence of sperm cells (oligozoospermic and azoospermic), immunoglobulin heavy constant gamma 2 (IGHG2) that was up-regulated in normozoospermic and aminopeptidase N (ANPEP), which was down-regulated in patient with low sperm motility (asthenozoospermic men). Then, western blot analysis for only one differentially expressed protein (ECM1) was performed in an independent set of samples to confirm these results (Table 2).

Saraswat et al. in an untargeted approach using shotgun proteomics compared the SP proteome from 7 normozoospermic and 10 asthenozoospermic men (Table 1) [34]. Samples were trypsin digested and analyzed by the ultra-performance liquid chromatography (UPLC)-MS (Table 1). They identified 429 proteins in SP samples. A label free strategy was performed to quantify these proteins, followed by statistical data analysis including principal component analysis and orthogonal projections to latent structures discriminant analysis (OPLS-DA), to identify the proteins significantly different among the two groups. Although some proteins were differentially expressed between the two groups, no statistical significance was found for seminal plasma dataset. Some of the upregulated and downregulated proteins in asthenozoospermic men are reported in Table 2.

Finally, using a shotgun approach, Liu et al. performed a quantitative proteomic strategy to identify and quantify the potential biomarkers of oligoasthenozoospermia [35]. SP samples from 10 men with oligoasthenozoospermia and 10 men with normozoospermia were separately pooled and enzymatically digested; the resulting peptides were labelled with isobaric Tags for relative and absolute quantification (iTRAQ) reagents and then analyzed by two-dimensional RP-RP-HPLC and MALDI-TOF/TOF MS (Table 1 and Table 2). The iTRAQ method utilizes isobaric tags to label peptides and proteins at the N-terminus and provides a multiplexing approach to compare up to eight different samples in a single run [67]. The authors performed 2D-HPLC, providing a separation orthogonality in the RP-RP system using pH 10 in the first and pH 3.0 in the second dimension, according to a method described early by Gilar et al. [68]. More than 700 seminal plasma proteins were both identified and quantified. The differential proteomic analysis revealed the downregulation of 20 proteins and the overexpression of 22 proteins in oligoasthenozoospermic patients in comparison to normozoospermic individuals. In particular, they identified CD177 antigen (CD177), prolactin-inducible protein; (PIP), PSA, lactotransferrin (LTF), prostaglandin-H2 D-isomerase (PTGDS), epididymal secretory protein E1 (HE1 or NPC2) and ECM1, as potential biomarkers of oligoasthenozoospermia (Table 2) [35]. Interestingly, for some of these proteins, i.e., NPC2 and ECM1, the results confirm the observations of previous studies [26,33], which described an overexpression of these species in fertile subjects compared to infertile ones, validating their utility as biomarkers of male fertility status.

### 3.2. SP Proteome Analysis by Top-Down Approach

The characterization of the human SP proteome by a top-down approach has been first explored by Fung and colleagues, describing the direct analysis of a pooled (*n* = 5) unfractionated SP sample by MALDI-TOF MS [24]. The strategy described by Fung et al., showing intact molecular features in a *m/z* range from 500 to 10.000, enabled the detection of endogenous peptides of SP in addition to multiple protein isoforms [24]. They also performed a comprehensive analysis of the peptide and protein constituents of the SP sample by combining classical 1D/2D gel electrophoresis with both MALDI-TOF and ESI-LC MS/MS, allowing the identification of over 100 different proteins [24].

Until now, only a few top-down investigations have been reported for the differential expression analysis of SP proteins between fertile and infertile patients (see Table 1 and Table 2) [36,37].

Very recently, our group has optimized a practical and efficient method for SP peptide enrichment before MALDI-TOF MS analysis, with the aim to reveal a diagnostic signature of male infertility [36]. Using a top-down strategy, we applied a dispersive-solid-phase extraction (d-SPE) coupled to MALDI-TOF MS to reveal SP peptides in their native and biologically active forms. In particular, commercially available octadecyl (C18)- and octyl (C8)-bonded silica sorbents and hexagonal mesoporous silica (HMS) were used to finely modulate the low molecular weight profiling of SP samples and best performances were obtained for C18-bonded silica. Finally, to assess the diagnostic potential of the platform, C18-bonded silica d-SPE and MALDI-TOF-MS were used to generate enriched endogenous peptide profiles from 15 fertile and 15 non-fertile donors and a key peptide-pattern within spectra was found to discriminate the two groups (Table 1 and Table 2). Seven differentially expressed peptides were identified, which were downregulated in the infertile patients compared to the fertile men. These peptides were fragments of SEMG I and SEMG II, which are abundant proteins in SP with key roles in coagulation and liquefaction processes. Interestingly, these findings are in contradiction with those of Sharma and others, who reported an augmented expression of SEMG I in oligoteratozoospermic patients as mentioned above [28]. On the other hand, a recent shotgun proteomics investigation by Martins and colleagues [39] reported both SEMG I and SEMG II to be under-expressed both in primary (inability to achieve pregnancy) and secondary infertile (inability to achieve pregnancy after at least one previous successful pregnancy) subjects compared to the fertile controls. It should be noticed that validation experiments performed by western blot only confirmed SEMG II decrease in primary infertility, while no change in the expression of both SEMG I and SEMG II was observed, again by western blot, in the secondary infertility group [39]. These apparently contradictory findings on semenogelins expression may derive from the intrinsic limitations of bottom-up approaches and by the presence of both intact semenogelins and peptide-derived semenogelins in SP. In fact, all the proteins are digested before LC-MS/MS analysis in a bottom-up approach. As a consequence, it is not possible to determine whether the peptides contributing to the identification of both SEMG I and II originated from intact precursors or from a fragmented protein. It is important to emphasize that a top-down strategy does not require the use of trypsin or more in general proteolytic digestion in comparison to bottom-up strategy. Our findings strongly consolidate the importance of semenogelins in male infertility (Table 2) [36].

Another top-down investigation which analyzed human SP proteins in association with the male fertility status was performed by Cadavid and colleagues using SELDI (surface-enhanced laser desorption/ionization)-TOF MS technology [37]. In SELDI-MS, analytes are applied to a protein chip array, which may be composed by different surfaces commonly used in chromatographic techniques (cationic, anionic, hydrophobic surfaces, etc.) or by biochemical bait molecules (immobilized antibodies, receptors) or also by DNA oligonucleotides. These surfaces are designed to retain proteins according to their chemical and physical characteristics (i.e., hydrophobic, hydrophilic, acidic, basic, metal affinity). It has been extensively used in proteomics studies, thanks to its high throughput, but suffers some drawbacks, including low resolution, poor reproducibility, both within and also between laboratories, and the inability to directly identify proteins because of the lack in MS/MS capabilities [69,70]. Cadavid and colleagues analyzed SP samples obtained from seven healthy fertile men and nine men with fertility alterations, including altered sperm progressive motility and sperm count (Table 1) [37]. Protein profiles of the SP samples were obtained by SELDI-TOF MS over a strong anion exchanger ProteinChip^®^ Q10 array. By performing ROC curves, they found 10 SP proteins statistically upregulated in the infertile group compared to the fertile one, though they did not finalize the identification of the proteins. Using the previously published database for seminal proteins [23,29] the authors hypothesized the potential identity of the differentially expressed proteins by using the *m/z* value obtained for each peak with differential expression between fertile and infertile subjects (see Table 2 for biomarkers). It is interesting to note that, in the case of statistically significant peaks in the low mass range (<20,000 Da), they were not able to propose the potential identity of some biomarkers or alternatively, in some cases, the attribution of putative identity appears forced considering the elevated mass error of such species [37]. One of the major limitations in fact, in SELDI mass spectrometer, is the lack of the MS/MS identification step, which precludes the possibility to assign the identity of potentially new endogenous peptides easily detectable by a top-down approach or to identify post-translational modifications and proteolytic products.

Additionally, the only other two investigations based on SELDI-TOF MS were used to assess protein changes in the SP of oligozoospermic [71] and non-obstructive azoospermic patients [72], although these data are not publicly available in an online database. In the first study, Yang and colleagues performed the differential analysis between the SP of fertile men and oligozoospermic patients by SELDI-TOF MS with H4 ProteinChip array surface (hydrophobic/reverse-phase array) and SAX-2 ProteinChip array surface (strong anionic exchanger array) [71]. The authors observed three differentially expressed protein peaks, that could be useful for screening potential oligozoospermic individuals [71].

In another study, Bai et al. analyzed and compared the SP proteome among non-obstructive azoospermic, severe oligozoospermic and fertile group by SELDI-TOF MS with the CM10 protein chip [72]. They stated that SP proteins compositions of severe oligozoospermic and healthy fertile men were similar, but both differed from non-obstructive azoospermic men [72].

## 4. Relevance of Pre-Analytical and Analytical Issues for SP Proteome Profiling and Identification of Male Infertility Biomarkers

The studies here reviewed reported the SP proteome profiling of infertile patients with quantitative and/or qualitative alteration of semen parameters. Although routine semen analysis is still the cornerstone for the clinical evaluation of male fertility/infertility, the exploding research on SP proteome could revolutionize the field of male infertility diagnosis and its clinical management. In fact, MS-based proteomics investigations on SP identified a plethora of potential biomarkers that could be useful for the non-invasive assessment of male reproductive conditions and for the differentiation of the various infertility etiologies. However, candidate markers identified in the here reviewed differential proteomic investigations were not always confirmed among studies.

Such discrepancies among different studies yet sharing patient cohorts with same infertility conditions, shed light on the challenges for restricting to a single or at most a few putative biomarkers for male infertility issues (see Table 2).

There are several pre-analytical and analytical factors which can influence data reproducibility, limiting the comparison of the here reviewed study results (Table 1). Such data variability could arise from many sources, among them differences in sample collection and processing, patient selection, analysis of individual or pooled samples, intra and inter-individual biological variations, MS techniques used for proteomic investigation and differences in proteomics strategies, the use or not of quantification methods, bioinformatics and the interpretation of collected data, etc. [73].

To date, the effects of different sample processing and the influence of both pre-analytical and analytical variables on SP proteomic profiling have not been extensively investigated in the proteomics studies which analyzed this specific biological fluid.

In the following sections, the effects of different sample processing and the influence of both pre-analytical and analytical variables on SP proteomic profiling will be discussed in relation to the here reported investigations.

### 4.1. Pre-Analytical Issues

The inherent features of SP, namely complexity and heterogeneity, pose many hurdles especially in comparative studies, which require standardized procedures for the normalization of MS data. It is well established that sample collection and processing methods significantly influence the mass spectra profile [74,75,76]; therefore, one of the major pre-analytical challenges in the proteomic analysis of SP is the lack of a standardized processing protocol. In fact, as pointed out in Table 1, SP specimens were obtained from semen samples with striking differences in the force, the number and the duration of centrifugation steps, in the timing and temperature of semen liquefaction. Specifically, the above reviewed investigations, indicated one [25,26,28,30,32,36] or more steps [27,29,31,33,34,35,37] for sample centrifugation at different force conditions (ranging from 500 to 100,000× *g*); some of them did not provide precious protocol details about timing of semen liquefaction [27,33,34,37]. Obviously, all these variables could contribute to the heterogeneity of SP analyzed by MS.

Furthermore, SP is characterized by a high dynamic range of protein abundance, with the top 10 most abundant proteins that account for about 80% of the total protein content [16,20,23]. The wide range of protein concentrations could mask the presence of low-abundance components, which may still play a key role in reproductive processes. To address these issues, the depletion and pre-fractionation of high abundance proteins as well as enrichment procedures of lower abundant ones should be applied [20]. However, also the use or not of sample separation or fractionation before analysis may result in high variation for protein detection and identification among different experiments and studies (Table 1). In fact, it is quite conceivable that various subproteomes in seminal plasma were extracted and converted into MS-profiles in those studies which used different chemical groups and chromatographic features for sample fractionation (Table 1).

Protease activity as well as proteins PTMs contribute to strongly increased SP protein complexity providing different variants detected in independent studies and data heterogeneity [73,77].

### 4.2. Intra and Inter-Individual Variability Related Issues

MS-based proteomics of SP may accelerate biomarker discovery of infertility by comparative differential proteomics in untargeted approaches. However, heterogeneity or small-sample size of fertile vs. infertile populations necessarily lead to differences in study results (Table 1 and Table 2).

In view of the intrinsic intra- and inter-individual variability of SP samples, larger cohorts would be necessary in order to compare the proteomes in the presence or absence of male infertility disorders. As a matter of fact, one of the main concerns in clinical proteomics remains to obtain a number of samples high enough to reach statistical significance. Hence, proteomic studies on sufficiently large cohorts of patients with standardized protocols are warranted to validate the preliminary markers identified in the clinical practice.

In the context of intra and inter-individual variability, pooling or not samples (see Table 1) might also have implications that should be taken into consideration during proteomic experimental design, in order to avoid some unforeseen methodological and statistical bias. In fact, although the biological variance among pools is reduced compared to that among individuals, proteins visible in individual samples are not always detectable when the pooling of samples are performed, with a potential loss of information due for example to dilution effects [78,79]. With the exception of the study by Drabovich and colleagues [25], which provided a complementary proteome analysis on pooled and individual samples, about half of the here reviewed studies performed only pooling of samples [27,28,30,31,33,35]. Sample pooling is preferably suitable for proteomic analyses when it is representative of the individual samples used to constitute the pool. Additionally, pooling samples could decrease the study power and modify the mean value or standard deviation of such analyte, and this could affect the value of statistical tests [78,79]. In summary, the choice to analyze individual or pooled samples is a crucial step due to its potential impact on the study design and consequently on the identification of reliable biomarkers of diseases.

### 4.3. Quantitation Issues

Concerning proteomics and peptidomics studies, the quest for quantitative strategies is stringently desirable in order to make data comparable among each other. MS-based quantitative proteomics can be divided into two main strategies: label-based and label-free. Although label-based methods provide an accurate and precise quantification, they have limitations, including the increased cost and sample preparation time, which limit the number of samples to be compared [80]. On the contrary, label-free strategies show a large dynamic range of quantification and allow for the quantitative comparison of different numbers of samples, reducing costs and the complexity of sample preparation [81].

### 4.4. Bottom-Up vs. Top-Down Approaches

It should be taken into account that low-abundant peptides and proteins, peptides derived from proteolytic cleavages and PTMs variants could be of elusive detection using shotgun proteomic approaches. The intrinsic limitations of such strategies, which require the use of trypsin or more in general proteolytic digestion before MS analysis, do not allow the detection of SP peptides and proteins in their native and biologically active forms. This may cause loss of information related to potentially important markers of male infertility.

Otherwise, top-down studies, which are well suited for the detection of naturally occurring peptides and small proteins, appear in a limited number (see Table 1 and Table 2). MS profiling strategies aiming at harvesting intact peptide signatures from SP samples may reveal one or more specific molecular patterns for the different infertility phenotypes.

It is important to notice that discordant results between reports could also derive from the different performances and detection capabilities of mass spectrometers which may affect spectral readouts. In fact, the use of ultra-high-resolution mass spectrometers or hybrid instruments with combined mass analyzers can achieve enhanced resolution, sensitivity and mass accuracy, increased dynamic range and fast acquisition rates with better qualitative and quantitative MS and MS/MS performances.

In light of the above considerations, in the next years the research focus should be directed towards the assessment of high-throughput MS-tools able to capture snapshots from SP containing diagnostic clinical information of infertility issues on an individual and population scale. Hence, there is an urgent need to identify a MS-based simple decision tree for the non-invasive differential diagnosis of infertility related diseases. Interestingly, seminal protein-based assays of TEX101 and ECM1 are under final validation for clinical use, providing the ability of MS to develop reliable clinical-grade assays [82,83,84]. In this scenario, it is worth noting that MALDI-MS has already become a routine laboratory diagnostic tool for the rapid, accurate and cost-effective identification of cultured bacteria and fungi in clinical microbiology, also proving to be capable of supporting screening and clinical decision-making [85,86,87].

Similarly, this high-throughput MS-technology may emerge, together with the clinically useful biomarkers discovered by proteomics, as a powerful diagnostic platform for translating the validated biomarkers of male infertility from bench to bedside. This could facilitate the development of drugs, devices, treatment options and especially new clinical-grade assays providing better management and care of infertile patients. Therefore, ‘top-down’ approaches should become necessary for valuable improvements in translational research exploiting their potential to revolutionize the field of diagnostics and therapeutics of male infertility associated scenarios.

### 4.5. Bioinformatic Interpretation of Collected Data

Careful attention should be paid in the appropriate interpretation of the huge amount of collected data, which could contribute to the discrepancies between reports from different authors.

The huge amount of scientific data generated during proteomic studies requires bioinformatic analysis using advanced software tools. The advancement in computational tools and user compatible analysis software provide a reliable interpretation of MS-based data.

Concerning issues related to bottom-up or top-down related MS data, most of data in spectral libraries is built from the in-silico enzymatic digestion of proteins, limiting the analysis only to peptides obtained from expected enzyme cleavage sites and also the number of PTMs considered; therefore, the datasets are difficult to leverage for peptidomics [88]. Moreover, the limited length of the aminoacidic sequences makes MS-peptidomics data analysis more challenging compared to bottom-up spectral data. The MS/MS patterns obtained from top-down approaches provide ‘non-tryptic’ peptides less informative than ‘tryptic digested ones’. In the light of the above considerations, the same bioinformatics strategies adopted for bottom-up approaches could not adequately perform in the case of less predictive and informative MS/MS spectra obtained from endogenous peptides [89]. Noteworthy, unlike bottom-up proteomics, software tools for peptidome characterization are not fully developed and MS data frequently requires skilled manual interpretation and rigorous validation [88,89]. De novo sequence algorithms together with classical database search provide sensitive and accurate peptide identification [90,91]. The application of software able to predict fragmentation patterns is one of most cost-effective ways to validate the identification [92].

More in general, the integration of proteomic and bioinformatic data provides insight into the function of proteins and peptides in cellular pathways. Hence, the advancement of infertility diagnostics and therapeutics depends on the integration of high-throughput “omics” data to identify accurate and specific biomarkers for infertility-related conditions. Network and pathway analysis using bioinformatic tools have been successfully used to obtain a wider picture on the putative pathways associated with the statistically significant biomarkers and their involvement in various infertility-related scenarios [16,93]. Most of the proteomics studies on SP used GO analysis to provide information about the localization, distribution and biological functions of the identified proteins. The integration of the datasets in male infertility still requires improved functionality, as currently few mathematical algorithms are available for cross omics data integration [94].

The integration of various proteomic datasets and bioinformatics databases should help to comprehensively annotate the biological functions and disease associations of the putative diagnostic markers of male infertility.

## 5. Concluding Remarks

Due to the presence of specific proteins secreted from organs of the reproductive tract, SP represents an excellent clinical diagnostic fluid for the discovery of biomarkers for the diagnosis of male infertility and their clinical translation.

It would be highly desirable that multi-analyte panels extrapolated by a high-throughput MS tool in an untargeted discovery phase might in future be able to reveal a distinctive pattern of molecular features correlated by specific relative expression to male fertility or infertility.

**Figure 3:**
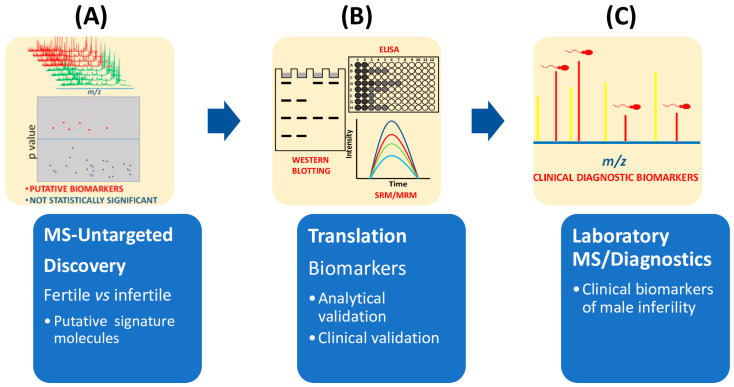
Flow chart of main steps required in clinical proteomics to select biomarkers of male infertility. (**A**) Untargeted MS-based proteomics approaches compare seminal plasma from fertile and infertile men. Mass data analysis, for example by multivariate statistical analysis, is used to extract the differences among mass spectra. (**B**) Potential biomarkers are then validated by different methodologies (immunohistochemistry, ELISA, immunoblotting) or mass spectrometry approaches such as SRM or MRM. (**C**) Before being approved as clinical biomarkers of infertility, clinical validation is also needed.

Currently, proteomics untargeted approaches mainly relying on the MS technological platform, provide not only the discovery of multiple biomarkers between fertile and unfertile populations (Figure 3), but also the accurate quantification of these signature molecules by MS [26,32,33,35]. However, the correct diagnosis of male infertility requires an accurate validation of putative signature molecules from the discovery phase (Figure 3). From the highlighted literature here reviewed, only a few studies have addressed this validation step [25,26,27,29,32,33,35]. Future developments for laboratory medicine diagnosis also require cheap MS instrumentation with ultra-fast high-throughput features as well as highly specialized and experienced personnel for the correct interpretation of the quality of the MS outputs.

It is important to notice that for what assisted reproduction technology (ART) is concerned, the major focus of proteomic investigations has been on the ejaculated spermatozoa. In fact, up to now, some differential proteomics investigations performed on semen have already identified putative biomarkers indicative of pregnancy outcome after ART [95,96,97,98,99]. In light of main findings from the here reviewed studies, which revealed how SP contains diagnostic clinical information of infertility issues, ART success rates may increase by placing the right focus on the critical role of SP proteome in fertilization. Therefore, further studies could be useful to clarify the role of SP in a successful pregnancy using ART.

Last, but not least, smart and fully automated software for time costs optimization and to further provide increasing accuracy might accelerate the application of MS in clinical laboratories.

## Figures and Tables

**Table 1:** Bottom-up and top-down MS-based proteomics studies on human SP.

Proteomic Approach	SP Processing and Fractionation	Samples Used for Proteomics	Normalization of Protein Quantitation	Quantification Strategies	Instrumentation/Techniques	Study Groups	References
Bottom-up	- 30 min at room temperature for semen liquefaction - 2000× *g* for 10 min at room temperature followed by 10,000× *g* for 20 min at 4 °C - No fractionation	58 individual samples	Yes	Label-free method (SpC ^1^)	1D-PAGE ^2^ ESI ^3^—Ion Trap/Orbitrap	- NZ ^4^ (*n* = 20) - AS ^5^ (*n* = 38)	Wang et al., 2009 [29]
Bottom-up	- 1 h at room temperature for semen liquefaction - 13,000× *g* for 15 min at room temperature (3 times) - RPLC ^6^ separation	- 30 individual samples-Pool of 5 NZ samples - Pool of 5 PV ^7^ samples - Pool of 5 Non-obstructive AZ ^8^ samples	Yes	Label-free method (SpC)	ESI-Triple-Quadrupole and Ion Trap/Orbitrap	- NZ (*n* = 12) - PV (*n* = 8) - Non-obstructive AZ (*n* = 10)	Drabovich et al., 2011 [25]
Bottom-up	- 2/3 h at room temperature for semen liquefaction - 13,000× *g* for 10 min at room temperature - SCX ^9^ -RPLC separation	Pooled samples for each group	Yes	Label-free methods (SpC and XIC ^10^)	ESI-Ion Trap/Orbitrap	- NZ (*n* = 5) - Non-obstructive AZ (*n* = 5) - PV (*n* = 5)	Batruch et al., 2012 [30]
Bottom-up	- 1 h (temperature not specified) for semen liquefaction - 16,000 rpm for 30 min at 4 °C (3 times) - RPLC separation	119 individual samples	Yes	Stable isotope dilution SRM ^11^	ESI-Triple-Quadrupole	- NZ (*n* = 42) - Non-obstructive AZ (*n* = 25) - Obstructive AZ (*n* = 10) - PV (*n* = 42)	Drabovich et al., 2013 [26]
Bottom-up	- <30 min (temperature not specified) for semen liquefaction - 15,000× *g* for 15 min at 4 °C followed by 100,000× *g* for 30 min at 4 °C - RP-RP HPLC ^12^ separation	Pooled samples for each group	Yes	Label-free method (SpC)	ESI-Ion Trap/Quadrupole/Orbitrap	- NZ (*n* = 11) - OAT ^13^ (*n* = 11)	Herwig et al., 2013 [31]
Bottom-up	- 20/60 min (temperature not specified) for semen liquefaction - 3000× *g* for 30 min - RP HPLC separation	Pooled samples for each group	Not mentioned	Label-free method (SpC)	ESI-Ion Trap	- NZ (*n* = 26) - TZ ^14^ (*n* = 22) - OZ ^15^ (*n* = 6) - OT ^16^ (*n* = 10)	Sharma et al., 2013 [28]
Top-down	- Time and temperature for semen liquefaction not specified - 2000× *g* for 15 min followed by 14,000× *g* for 10 min at 4 °C - SAX ^17^ separation	16 individual samples	Yes	Label-free method (peak intensities)	SELDI-TOF ^18^ MS	- NZ (*n* = 7) - OA ^19^ (*n* = 9)	Cadavid et al., 2014 [37]
Bottom-up	- Time and temperature for semen liquefaction not specified - 800× *g* (time not specified) followed by 14,000× *g* for 30 min at 4 °C - RP UPLC ^20^ separation	Pooled samples for each group	Yes	Label-free method (iBAQ ^21^)	ESI- Quadrupole/Orbitrap	- Adolescents without varicocele (*n* = 23) - Adolescent with varicocele and normal semen analysis (*n* = 37) - Adolescents with varicocele and altered semen analysis (*n* = 17)	Del Giudice et al., 2016 [27]
Bottom-up	- Time and temperature for semen liquefaction not specified - 2000× *g* for 20 min at 4 °C followed by 10,000× *g* for 20 min - RP UPLC separation	17 individual samples	Yes	Label-free method	UPLC-MS	- NZ (*n* = 7) - AS (*n* = 10)	Saraswat et al., 2017 [34]
Bottom-up	- 30 min (temperature not specified) for semen liquefaction - 1000× *g* for 15 min followed by 13,000× *g* for 10 min (temperature not specified) - RP-RP HPLC separation	Pooled samples for each group	Yes	Label-based method (iTRAQ ^22^)	MALDI-TOF/TOF ^23^ MS	- NZ (*n* = 10) - OA (*n* = 10)	Liu et al., 2018 [35]
Bottom-up	- 30 min at 37 °C for semen liquefaction - 40,000× *g* for 30 min at 4 °C - RPLC separation	6 individual samples	Yes	Label-based (TMT ^24^) and label-free methods (iBAQ)	ESI-Ion Trap/Orbitrap	- NZ (*n* = 3) - AS (*n* = 3)	Wu et al., 2019 [32]
Bottom-up	- Time and temperature for semen liquefaction not specified - 500× *g* for 10 min followed by 1500 × *g* for 10 min - RP HPLC separation	Pooled samples for each group	Yes	Label-based method (TMT)	ESI-Ion Trap/Orbitrap	- NZ (*n* = 4) - AS (*n* = 4) - OZ (*n* = 4) - AZ (*n* = 4)	Barrachina et al., 2019 [33]
Top-down	- 15/30 min at 37 °C for semen liquefaction - 15,000× *g* for 15 min at 4 °C - C_18_-bonded silica *d*-SPE ^25^	30 individual samples	Yes	Label-free method (peak intensities)	MALDI-TOF/TOF MS	- NZ (*n* = 15) - Infertile men (AT ^26^ = 2; OAT = 5; TZ = 6; OZ = 2)	Correnti et al., 2022 [36]

^1^ SpC, spectral counting. ^2^ 1D-PAGE, one dimensional-polyacrylamide gel electrophoresis. ^3^ ESI, electrospray ionization. ^4^ NZ, normozoospermic. ^5^ AS, asthenozoospermic. ^6^ RPLC, reversed-phase liquid chromatography. ^7^ PV, post-vasectomy (simulates obstructive AZ). ^8^ AZ, azoospermic. ^9^ SCX, strong cation exchange. ^10^ XIC, extracted ion chromatograms. ^11^ SRM, selective reaction monitoring. ^12^ HPLC, high performance liquid chromatography. ^13^ OAT, oligoasthenoteratozoospermic. ^14^ TZ, teratozoospermic. ^15^ OZ, oligozoospermic. ^16^ OT, oligoteratozoospermic. ^17^ SAX, strong anion exchange. ^18^ SELDI-TOF, surface-enhanced laser desorption/ionization- time-of-flight. ^19^ OA, oligoasthenozoospermic. ^20^ UPLC, ultra-performance liquid chromatography. ^21^ iBAQ, intensity-based absolute quantification. ^22^ iTRAQ, isobaric tags for relative and absolute quantification. ^23^ MALDI-TOF/TOF, matrix-assisted laser desorption ionization- time-of-flight/time-of-flight. ^24^ TMT, tandem mass tag. ^25^ *d*-SPE, *dispersive*-solid phase extraction. ^26^ AT, asthenoteratozoospermic.

**Table 2:** MS-based profiling studies on human SP: major proteomics findings and candidate biomarkers of male infertility disorders using bottom-up and top-down approaches.

References	Study Groups	Main Findings	Candidate Biomarkers Expression Levels	Validation Assay
Wang et al., 2009 [29]	- NZ ^1^ (*n* = 20) - AS ^2^ (*n* = 38)	45 proteins were ↑ ^3^ and 56 proteins were ↓ ^4^ in the AS group. Most of these proteins originated from the epididymis and prostate. ITLN1, ADH, ALAD, DJ-1 were suggested as biomarkers for oxidative stress in AS patients	- ITLN1, ADH and ALAD were ↑ in AS. - DJ-1 was ↓ in AS.	Western Blot (DJ-1)
Drabovich et al., 2011 [25]	- NZ (*n* = 12) - PV ^5^ (*n* = 8) - Non-obstructive AZ ^6^ (*n* = 10)	18 biomarkers were identified at differential abundance between NZ, Non-obstructive AZ, and PV. Some testis-specific proteins (LDHC, TEX101, and SPAG11B) performed with absolute or nearly absolute specificities and sensitivities.	- TEX101, LDHC, SPAG11B, PTGDS, MUC15, FAM12B were ↓ in PV and Non-obstructive AZ	Stable-isotope dilution SRM ^7^
Batruch et al., 2012 [30]	- NZ (*n* = 5) - Non-obstructive AZ (*n* = 5) - PV (*n* = 5)	- 34 proteins were ↑ in NZ compared to Non-obstructive AZ men. - 18 were ↓ in NZ compared to Non-obstructive AZ. - 59 were ↑ in Non-obstructive AZ compared to PV patients. - 16 were ↓ in Non-obstructive AZ compared to PV patients. Many of these proteins are linked to fertility and are involved in sperm capacitation, spermatogenesis and intracellular signaling.	- STOM, OVCH2, PTGDS, CRISP2, LIPI, LDHC, SERPINA6, CA4, HIST1H2BA, MPO were ↓ in Non-obstructive AZ compared to NZ - VAV2, TGM2, SPARC, KIAA0368, EPS8L2, SPARCL1, COL6A2, DDX1, CST2, CST4 were ↑ in Non-obstructive AZ compared to NZ - MUC5B, CPVL, CRIM2, SLLC2A5, ELSPBP1, PATE4, LOC642103, SPINT3, COL18A1, BGN were ↑ in Non-obstructive AZ compared to PV - HIST1H2BL, FGG, AZU1, MPO, GSTM2, PRELP, ORM1, FLJ11151, FGB, PAEP were ↓ in Non-obstructive AZ compared to PV	None
Drabovich et al., 2013 [26]	- NZ (*n* = 42) - Non-obstructive AZ (*n* = 25) - Obstructive AZ (*n* = 10) - PV (*n* = 42)	Epididymis-expressed ECM1 and testis-expressed TEX101 were differentially expressed between Obstructive AZ and Non-obstructive AZ patients and may serve as biomarkers of azoospermia	- ECM1 was ↑ in NZ and Non-obstructive AZ and ↓ in Obstructive AZ men - TEX101 was ↑ in NZ and ↓ in Non-obstructive AZ and Obstructive AZ men	ELISA (ECM1) Immunohistochemistry analysis (TEX101) Immuno-SRM assay (TEX101)
Herwig et al., 2013 [31]	- NZ (*n* = 11) - OAT ^8^ (*n* = 11)	24 proteins were ↑ in the OAT compared to NZ men. These proteins are mainly involved in metabolism and inflammation, defense, and stress responses	AACT and ALDR were ↑ in OAT.	None
Sharma et al., 2013 [28]	- NZ (*n* = 26) - TZ ^9^ (*n* = 22) - OZ ^10^ (*n* = 6) - OT ^11^ (*n* = 10)	20 proteins were differentially expressed among the 4 groups. Biological regulation is the main process affected.	- MUC6, ORM1 precursor and AEG-like isoform 1 precursor were ↓ in TZ - CLU isoform 1 was ↓ in the OZ group - PAP was ↓ in TZ and OZ - PSA isoform 1 preprotein, SEMG I isoform b preprotein were ↑ in OT - CST3 was ↓ in OT - ZA2G 1 and TIMP-1 precursor were ↑ in OZ - PTPRS isoform 1 precursor was ↑ in TZ - DJ-1 was ↑ in TZ and OZ	None
Cadavid et al., 2014 [37]	- NZ (*n* = 7) - OA ^12^ (*n* = 9)	Ten proteins were identified with a statistical difference between the NZ and OA patients	UBE2C, CSTA, DCD, GNAO, IDE, CERU, SLIT2 Isoform 1, UGT 1 precursor, IQGAP1 were ↑ in OA	None
Del Giudice et al., 2016 [27]	- Adolescents without varicocoele (*n* = 23) - Adolescent with varicocele and normal semen analysis (*n* = 37) - Adolescents with varicocele and altered semen analysis (*n* = 17)	- Cab45, protein lefty-1, DNase I, PAP2-alpha were suggested as biomarkers of spermatogenesis and homeostasis associated to the control group. - IBP-7, HDC, and CRISP-3 were suggested as biomarkers associated to varicocoele. These proteins participate in cell adhesion, immune and defense response.	- Cab45 was ↓ in varicocoele groups - CRISP-3 was ↑ in Adolescents with varicocoele and altered semen analysis.	Western Blot (Cab45 and CRISP-3)
Saraswat et al., 2017 [34]	- NZ (*n* = 7) - AS (*n* = 10)	429 proteins were differentially expressed between NZ and AS	- WAC, RANB3, TICN3, PGAM4, DI3L1, DPP3, MP2K5, TERA, TEX14, SLTM were ↓ in AS - SPON2, BSPH1, LX15B, PRDX5, MRO2A, PP1B, IL1FS, A1BG, EFCB5 were ↑ in AS	None
Liu et al., 2018 [35]	- NZ (*n* = 10) - OA (*n* = 10)	22 proteins were ↑ and 20 proteins were ↓ in the OA patients. These proteins were involved in physiological processes, including metabolism, transport, antioxidation and immune response.	- PSA, ECM1, HE1, PTGDS and CD177 were ↓ in OA. - LTF and PIP were ↑ in OA.	Western Blot (PSA and LTF)
Wu et al., 2019 [32]	- NZ (*n* = 3) - AS (*n* = 3)	29 proteins were differentially expressed between the two groups: - 22 proteins were ↑ in NZ group - 7 proteins were ↓ in NZ group. Most of these proteins are mainly associated with sperm motility, spermatogenesis and male infertility	- SORD, ANXA2 and KLK2 were ↓ in AS. - HSPA2 was ↑ in AS.	Western Blot (KLK2, HSPA2, SORD, ANAX2)
Barrachina et al., 2019 [33]	- NZ (*n* = 4) - AS (*n* = 4) - OZ (*n* = 4) - AZ (*n* = 4)	6 proteins were differentially expressed among the groups. These proteins are involved in sperm-oocyte binding, fertilization and immune response.	CRISP1, NPC2, SPINT3, and ECM1 were ↓ in OZ and AZ IGHG2 was ↑ in NZ ANPEP was ↓ in AS	Western Blot (ECM1)
Correnti et al., 2022 [36]	- NZ (*n* = 15) - Infertile men (AT ^13^ =2; OAT = 5; TZ = 6; OZ = 2)	7 peaks were statistically different between NZ and infertile men.	All the peaks were ↓ in infertile men compared to NZ: - *m/z* = 2331 (Fragm: 330–349; SEMG I) - *m/z* = 2362 (Fragm: 248–267; SEMG II) - *m/z* = 2482 (Fragm: 195–215; SEMG I) - *m/z* = 2893 (Fragm: 428–453; SEMG I) - *m/z* = 3059 (Fragm: 248–273; SEMG I) - *m/z* = 3083 (Fragm: 248–273; SEMG II) - *m/z* = 3938 (Fragm: 549–582; SEMG II)	None

^1^ NZ, normozoospermic. ^2^ AS, asthenozoospermic. ^3^ **↑**, upregulated. ^4^ **↓**, downregulated. ^5^ PV, post-vasectomy (simulates obstructive AZ). ^6^ AZ, azoospermic. ^7^ SRM, selective reaction monitoring. ^8^ OAT, oligoasthenoteratozoospermic. ^9^ TZ, teratozoospermic. ^10^ OZ, oligozoospermic. ^11^ OT, oligoteratozoospermic. ^12^ OA, oligoasthenozoospermic. ^13^ AT, asthenoteratozoospermic.

## Data Availability

Not applicable.
